# Case report: Long-term intracranial effect of zimberelimab monotherapy following surgical resection of high PD-L1-expressing brain metastases from NSCLC

**DOI:** 10.3389/fonc.2024.1390343

**Published:** 2024-05-10

**Authors:** Weijia Wu, Jinyou Guo, Lianxiang He, Qi Deng, Xianping Huang

**Affiliations:** ^1^ Department of Cardiothoracic Surgery, The Second Affiliated Hospital of Wenzhou Medical University, Wenzhou, Zhejiang, China; ^2^ Department of Oncology, Yuhuan Second People’s Hospital, Yuhuan, China; ^3^ Department of Medical Affairs, Guangzhou Gloria Bioscience Co., Ltd., Beijing, China

**Keywords:** brain metastases, NSCLC, PD-L1 positive, immunotherapy, PD-1

## Abstract

Non-small cell lung cancer (NSCLC) accounted for the majority of lung cancer cases worldwide. Brain metastases (BM) frequently complicate NSCLC and portend a dismal prognosis. To control neurological symptoms, surgical resection is commonly followed by brain radiotherapy (RT). However, RT is often complicated by neurotoxicity. For patients with tumors that harbor positive driver genes, tyrosine kinase inhibitors are considered the standard of care. Nevertheless, treatment options for those without driver gene mutations are still debated. Programmed death receptor 1 (PD-1)/ligand 1 (PD-L1) inhibition has emerged as a novel therapeutic strategy for NSCLC patients with PD-L1-positive tumors, as well as for those with asymptomatic BM. However, the effect of anti-PD-1 antibodies on active BM within such specific populations is undetermined. Herein we present a case of a 65-year-old patient with NSCLC and high PD-L1-expressing BM. The patient underwent surgical resection of BM followed by first-line monotherapy with 31 cycles of zimberelimab, a novel anti-PD-1 antibody, and has already achieved 24 months of progression-free survival and intracranial recurrence-free survival. To our knowledge, this is the first report regarding the intracranial effect of zimberelimab on BM from primary lung cancer. This case report might facilitate an understanding of the intracranial effects of different anti-PD-1 antibodies for such populations.

## Introduction

Lung cancer is the second most prevalent cancer worldwide, resulting in more than 2 million new cases and approximately 1.8 million deaths during 2020 (1), and non-small cell lung cancer (NSCLC) accounted for over 80% of all lung cancer cases (2). Brain metastases (BM) are present in approximately 10% of patients at initial diagnosis, and eventually afflict 20-40% (3). The prognosis for patients with BM from NSCLC is extremely unfavorable, with a median survival of less than 6 months in untreated individuals (4–6). Age, extracranial tumor activity, the number of BM lesions, and the initial tumor type/molecular subtype play pivotal roles in determining patient outcomes (1, 7).

Current treatment options for BM include stereotactic radiosurgery (SRS) alone for patients with NSCLS and limited BM, and surgical resection followed by SRS or whole brain radiotherapy (WBRT) for selected patients with symptomatic BM or for diagnostic purposes (8). However, radiotherapy (RT) is complicated by neurotoxicity in up to 35% of cases (9–13), particularly among patients with large tumors, typically defined as those with diameters greater than 2 cm (14). Moreover, chemotherapy is administrated infrequently due to its well-known limited penetration of the blood-brain barrier (BBB) (15). For patients with NSCLC harboring positive driver genes, tyrosine kinase inhibitors are considered the standard of care. Nevertheless, optimal therapeutic approaches for patients with intracranial metastases from NSCLC, especially those lacking driver gene mutations, are still being debated.

Immune checkpoint inhibitors (ICIs) that target programmed death receptor 1 (PD-1)/ligand 1 (PD-L1) have emerged as the most promising treatment approach and have been approved for first-line treatment of NSCLC with high PD-L1 expression (Tumor Proportion Score (TPS) ≥ 50%) and without EGFR mutations or ALK/ROS1 alterations. Furthermore, accumulating evidence shows that anti-PD-1 antibodies may induce durable responses in patients with asymptomatic BM, challenging the long-held notion that the central nervous system is an immunologic sanctuary site (16–19). However, the role of immunotherapy on active BM in selected populations is uncertain as most clinical trials have excluded untreated or symptomatic patients. Additionally, whether anti-PD-1 monotherapy following surgical resection of BM with high PD-L1 expression may prolong the time to intracranial recurrence has not been investigated.

To the best of our knowledge, we present the first case of a patient with NSCLC and high PD-L1-expressing BM (TPS = 95%) who received zimberelimab, a PD-1 inhibitor, as first-line monotherapy following surgical resection of BM. Remarkably, by the end of March 2024, this patient had achieved a durable tumor response with intracranial recurrence-free survival (RFS) exceeding 24 months while maintaining an unaffected quality of daily life.

The PD-1 inhibitor used in our case was zimberelimab, a novel fully humanized anti-PD-1 monoclonal immunoglobulin G4 developed from the OmniRat transgenic platform (20, 21). Compared with another PD-1 inhibitor (pembrolizumab, not in a head-to-head comparison), zimberelimab achieved a higher overall response rate (ORR) in relapsed/refractory classical Hodgkin lymphoma in a phase II clinical trial (22, 23), and is approved in China for this indication (24). Furthermore, based on the encouraging outcomes of a pivotal phase II clinical trial, zimberelimab has recently obtained approval for the treatment of cervical cancer and stands as the first and only anti-PD-1 antibody approved for this indication in China, with a remarkable ORR of 27.6%, unparalleled within its therapeutic class (25).

## Case report

In January 2022, a 65-year-old male was admitted to the Department of Neurosurgery at our institution for the evaluation of subjective right-sided extremity weakness. His symptoms had persisted for two weeks and had progressed acutely on the day before admission. He denied having fever, chest tightness, shortness of breath, nausea, or vomiting. He had a ten-year history of pulmonary tuberculosis which had not been treated regularly, and was being treated with levofloxacin and linezolid at the time of admission.

**Figure 1:**
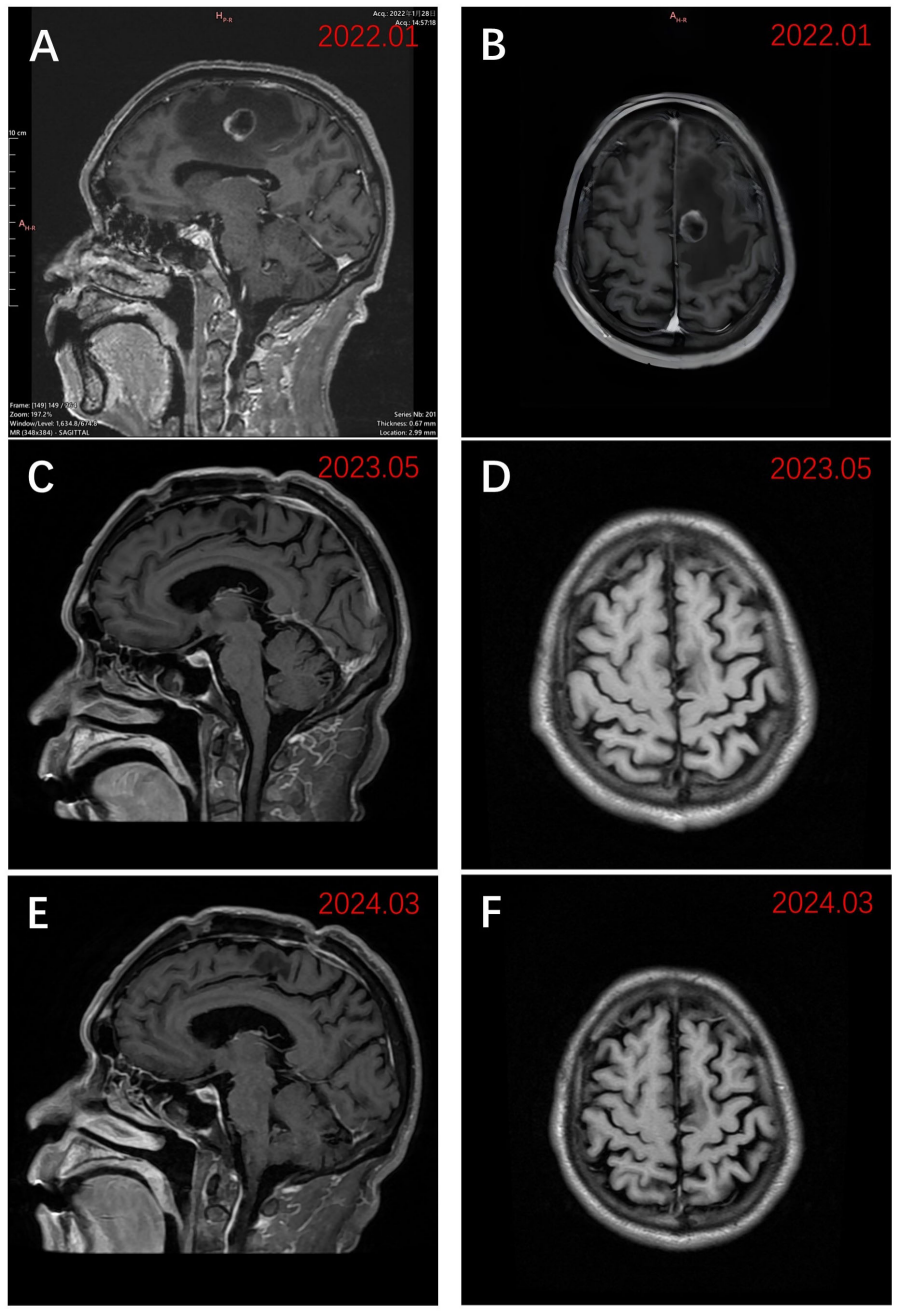
Sagittal and axial views of MRI images using T1WI enhancement. **(A)** Preoperative sagittal view in late January 2022 demonstrated a 20 × 19 mm lesion of the left parietal lobe; **(B)** Preoperative axial view in late January 2022; **(C)** Sagittal view in early May 2023 after receiving zimberelimab; **(D)** Axial view in early May 2023 after receiving zimberelimab. **(E)** The most recent sagittal view (March 2024); **(F)** The most recent axial view (March 2024).

**Figure 2:**
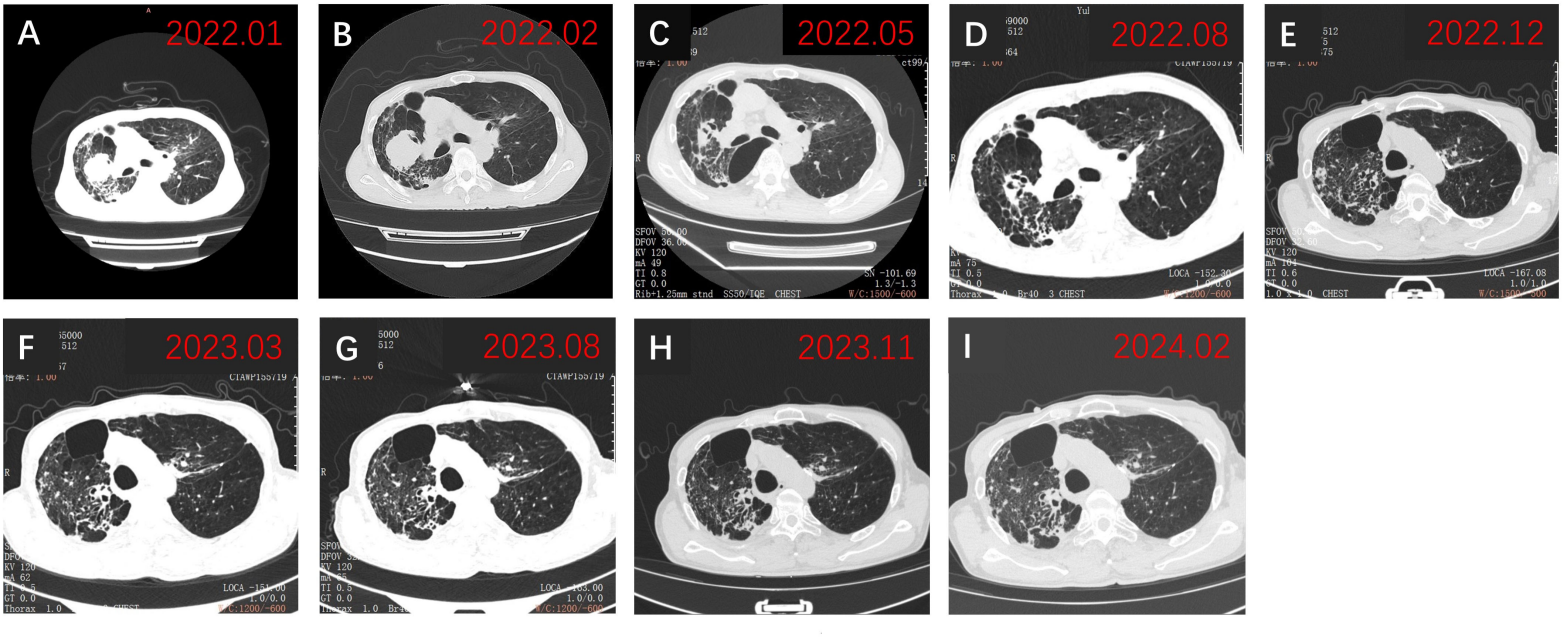
Chest CT findings. **(A)** Chest CT revealed a tumor on admission in January 2022; **(B)** Chest CT showed persistent abnormalities before initiation of zimberelimab in February 2022; **(C)** Chest CT demonstrated a significant decrease in lesion size, achieving PR in May 2022; **(D-I)** Subsequent chest CTs showed sustained PR of the tumor. PR, partial response.

Physical examination revealed a blood pressure of 114/96 mmHg, heart rate of 102/min, respiratory rate of 20/min, left limb muscle strength of grade 5, right limb muscle strength of grade 4, with normal muscle tone. His Eastern Cooperative Oncology Group performance status score was assessed at 1. Enhanced magnetic resonance imaging (MRI) of the brain exhibited a 20 × 19 mm circular abnormal signal shadow within the medial left parietal lobe (**Figures 1A, B**). Chest computed tomography (CT) revealed a mass in the upper lobe of the right lung, along with multiple satellite lesions (**Figure 2A**). We assessed that the patient’s progressive subjective right-sided weakness with only slight hemiparesis was probably caused by the location of the lesion in the parietal somatosensory cortex.

**Figure 3:**
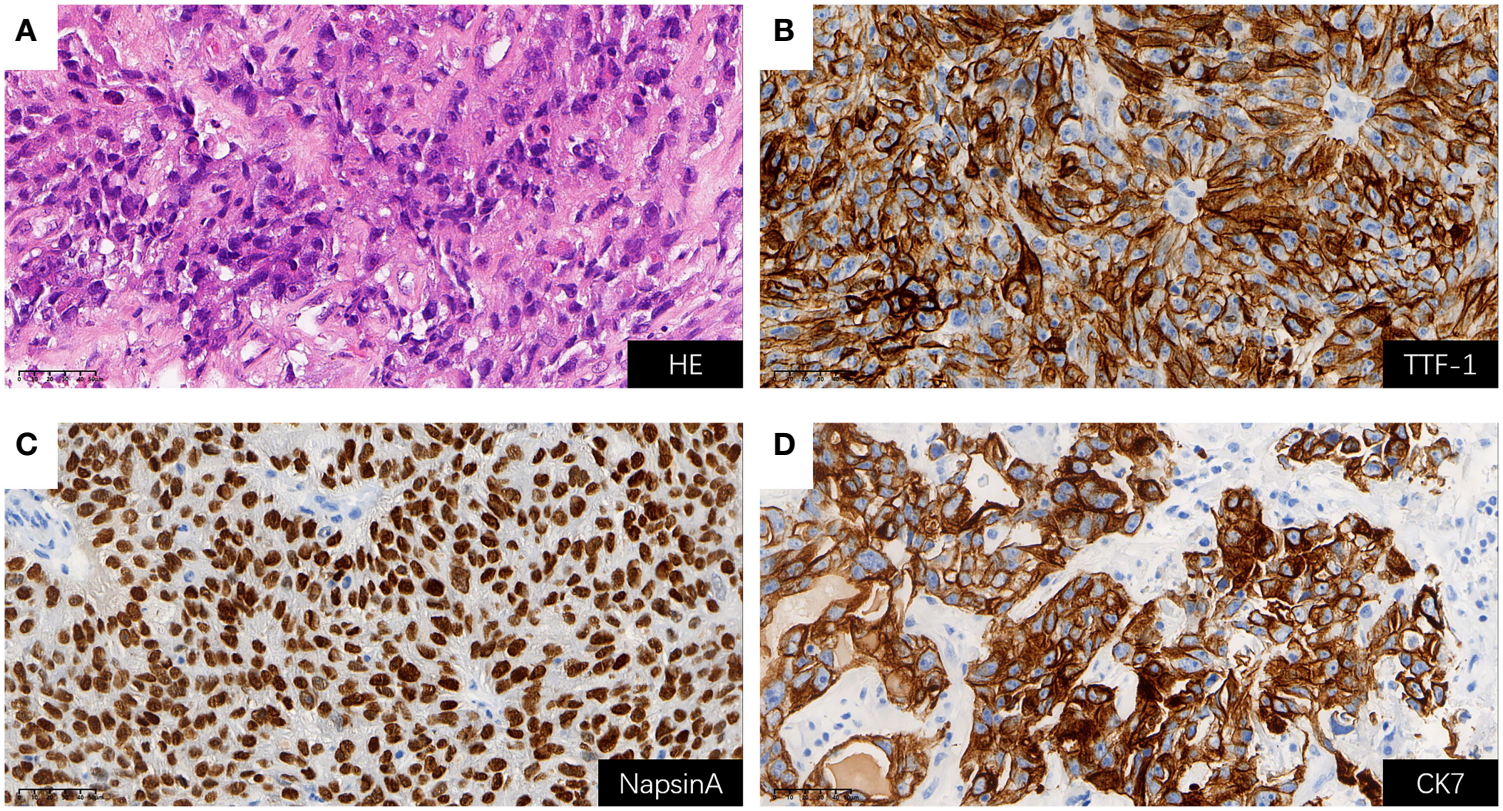
H&E staining and positive Immunohistochemical staining results for BM (all shown at 400× magnification); **(A)** H&E staining; **(B)** TTF-1 (+); **(C)** NapsinA (+); **(D)** CK7 (+). BM, brain metastasis; H&E, hematoxylin and eosin.

**Figure 4:**
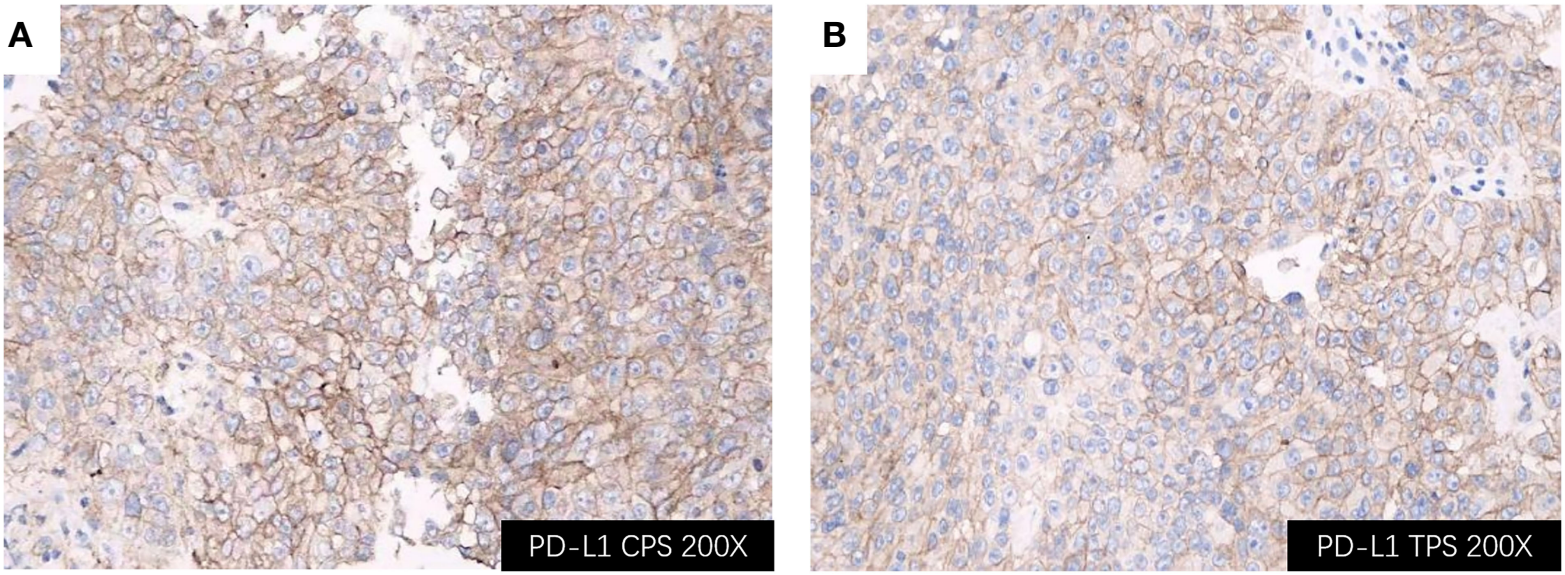
Immunohistochemical staining of the BM lesion revealed positivity for PD-L1 (all shown at 200× magnification); **(A)** PD-L1 CPS of 98; **(B)** PD-L1 TPS of 95%. BM, brain metastasis; PD-L1, programmed cell death ligand 1; CPS, Combined Proportion Score; TPS, Tumor Proportion Score.

Considering rapidly progressive neurological symptoms and an objective finding of right-sided hemiparesis from suspected active BM of lung cancer, the patient underwent neuronavigation-guided intracranial tumor resection and cranioplasty. Histopathologic examination confirmed the diagnosis of metastatic pulmonary adenocarcinoma. Immunohistochemical staining results (**Figure 3**) yielded the following results: CK (+); CK7 (+); TTF-1 (+); NapsinA (+); CDX-2 (-); Villin (-); EMA (+); PR (-); E-cad (+); SSTR2 (-); GFAP (-); S-100 (-); Ki-67 (about 60%+ in hot-spot region); GATA-3 (-); SALL4 (-); STAT6 (-). Tumor PD-L1 expression testing (clone 22C3) of the BM displayed a combined positive score (CPS) of 98 and a TPS of 95% (**Figure 4**). The tumor harbored neither EGFR gene mutation nor ALK gene rearrangement. The patient received a diagnosis of metastatic NSCLC (stage IVA) without gene variants.

Three weeks after BM resection, in late February 2022, the size of the right lung lesion was measured as 47 mm × 45 mm by chest CT. (**Figure 2B**). Because systemic therapies rather than surgical excision are indicated for the treatment of primary lesions in stage IV NSCLC and considering the extremely high TPS of 95%, monotherapy with zimberelimab, a PD-1 inhibitor, was administered intravenously at a dosage of 240 mg every three weeks. In May 2022, after four cycles of therapy, the size of the pulmonary lesion was significantly reduced to 27 × 21 mm (**Figure 2C**), indicating a partial response (PR). Brain MRI showed no recurrent BM (**Figures 1C, D**). Zimberelimab was discontinued after a course of 31 cycles from February 2022 to January 2024, eventually reaching a two-year treatment duration. The patient remains in remission with a normal quality of life. As of the latest follow-up in March 2024, the patient has achieved both progression-free survival (PFS) and intracranial RFS of 24 months (**Figures 1E, F**, **2D-I**). This report does not include individually identifiable health information or photographs. The patient provided written informed consent for the publication of this article.

## Discussion

We present a patient with NSCLC and symptomatic BM characterized by high PD-L1 expression who achieved long PFS and intracranial RFS after BM resection followed by zimberelimab monotherapy instead of cranial RT. Although numerous clinical trials have explored the efficacy of ICI monotherapy in NSCLC patients with TPS ≥ 50%, these studies have predominantly enrolled highly selected individuals with stable or asymptomatic BM. Hence, limited data are available on the effect of ICIs in patients with active BM, particularly in reducing intracranial recurrence following BM resection.

The recommended treatment for active BM of NSCLC is BM tumor resection followed by WBRT to reduce the risk of local relapse and to protect distant brain tissue. However, due to the subsequent cognitive decline caused by WBRT, this technique has been replaced gradually by SRS, which aims to attain superior local control (26, 27), and results in a favorable median overall survival (OS) exceeding 12 months while mitigating the risk of neurocognitive complications of WBRT (28, 29). Nonetheless, a subset of elderly individuals cannot tolerate the adverse effects of SRS. Moreover, in addition to delaying neurological deterioration, another primary objective in the treatment of BM is to improve quality of life. Therefore, the exploration of alternative therapeutic options for BM of NSCLC is still important. Surgical excision of the primary NSCLC lesion is not indicated for stage IV disease (except for palliation of complications such as hemoptysis or bronchial obstruction); the mainstays of therapy comprise systemic treatments that include but are not limited to chemotherapy, ICIs, EFGR tyrosine kinase inhibitors, and monoclonal antibodies. Several studies (30, 31) demonstrated that PD-1/L1 inhibitors were more effective than chemotherapy in advanced NSCLC with TPS ≥ 50%. Considering the patient’s high TPS of 95% and the infeasibility of primary tumor resection, we administered zimberelimab monotherapy to control both the primary lesions and BM recurrence.

PD-1/PD-L1 inhibition has recently emerged as a prominent therapeutic strategy in oncology and has been utilized for the management of a variety of malignancies. ICI monotherapy has become an integral component of treatment for patients with NSCLC without driver mutations but with ≥50% expression of PD-L1 (32).

A few phase III clinical trials have investigated the efficacy and safety of PD-1/PD-L1 inhibitors in patients with high PD-L1-expressing (TPS ≥ 50%) NSCLC (33–35). These studies demonstrated that compared to standard combination chemotherapy, PD-1/PD-L1 inhibitors significantly prolonged median OS and median PFS.

A pooled analysis of KEYNOTE-001, KEYNOTE-010, KEYNOTE-024, and KEYNOTE-042 compared the effects of pembrolizumab versus chemotherapy in patients with PD-L1-positive advanced or metastatic NSCLC. Pembrolizumab demonstrated superior efficacy (PFS and OS) and activity (ORR and duration of response) compared to chemotherapy, irrespective of the presence of treated, stable BM at baseline (36), further supporting the use of PD-1/PD-L1 inhibitors in this population (37, 38).

However, patients with active or untreated BM were excluded from all these studies; consequently, the intracranial effect of anti-PD-(L)1 antibodies remains unclear. To date, data regarding the BBB penetration of ICIs are limited. Although conventional belief held that monoclonal antibodies are too large to penetrate the BBB, a recent study demonstrated that post-treatment cerebrospinal fluid levels of the anti-PD-1 antibody nivolumab in melanoma patients attained the half maximal effective concentration, suggesting that nivolumab can directly cross the BBB and exert intracranial activity (39). Additionally, a few reports suggested that pembrolizumab benefited patients with BM of NSCLC (15, 40, 41). These results may be explained by the effect of cytotoxic T cells, which are activated by ICIs in extracranial tumors and are also capable of crossing the BBB to destroy intracranial tumor cells (42).

To date, the strongest evidence of the efficacy of ICI therapy of active BM came from a phase II trial of pembrolizumab by Goldberg et al. The study included 42 patients with advanced non-oncogene-driven NSCLC and untreated asymptomatic BM (5–20 mm). Patients with PD-L1 ≥1% experienced an intracranial ORR of 29.7% (11/37) (seven patients exhibited PR on subsequent imaging, whereas four achieved complete response), with a median OS of 9.9 months (cohort 1), whereas BM responses of patients with PD-L1 < 1% were either absent or unevaluable (cohort 2) (15). Several analyses and retrospective studies have consistently validated these intracranial outcomes of pembrolizumab therapy, disclosing intracranial ORRs of 15 to 35% irrespective of PD-L1 expression; however, the intracranial ORR surpasses 50% in patients with PD-L1 ≥ 50% (43–46). Additionally, a retrospective study compared the intracranial and overall efficacies of first-line ICI-based therapy versus chemotherapy in patients with non-squamous NSCLC and BM, regardless of the receipt of prior RT. Median OSs were 7.6 months (95% CI: 4.6–20.0) and 22.8 months (95% CI: 8.8-NR), and median intracranial PFSs were 4.5 months (95% CI: 4.0–7.0) and 5.0 months (95% CI: 3.0-NR) in the chemotherapy and ICI groups, respectively. Notably, this study included patients who are typically excluded from clinical trials, especially the 25.4% of patients with a performance status ≥ 2. This finding suggests that certain patient populations, such as the elderly, may derive greater benefits from ICI therapy (47).

Although some studies demonstrated that a few anti-PD-1 antibodies might exert activity against BM, the question of whether efficacy differs between specific anti-PD-1 antibodies is still to be answered. The present case might strengthen our understanding of this issue. Recently, a similar case of a female with cervical cancer and BM was successfully treated with zimberelimab following RT (48). In addition, our experience suggests that anti-PD-1 immunotherapy may be adopted to obviate the neurotoxicity of postoperative WBRT. This hypothesis should undergo empiric evaluation in preclinical and potentially in clinical trials.

## Conclusion

While several clinical trials have investigated the efficacy of ICIs in patients with NSCLC exhibiting high TPS, limited data are available regarding their effectiveness in individuals with active BM. Here, we report the first case of symptomatic BM of NSCLC with high PD-L1 expression who underwent BM resection followed by zimberelimab monotherapy, and gained long-term intracranial RFS. Our patient’s response suggests that the individualized use of zimberelimab monotherapy may be considered in clinical practice and should be validated by prospective clinical trials.

## Data availability statement

The original contributions presented in the study are included in the article/supplementary material. Further inquiries can be directed to the corresponding author.

## Ethics statement

Institutional ethical approval is not deemed necessary for this case report. The studies were conducted in accordance with the local legislation and institutional requirements. The participants provided their written informed consent to participate in this study. Written informed consent was obtained from the individual(s) for the publication of any potentially identifiable images or data included in this article.

## Author contributions

WW: Data curation, Investigation, Writing – review & editing. JG: Data curation, Investigation, Writing – review & editing. LH: Writing – review & editing. QD: Writing – original draft. XH: Supervision, Writing – review & editing.
